# Nausea, vomiting and poor appetite during pregnancy and adverse birth outcomes in rural Nepal: an observational cohort study

**DOI:** 10.1186/s12884-020-03141-1

**Published:** 2020-09-17

**Authors:** Amanda Regodón Wallin, James M Tielsch, Subarna K Khatry, Luke C Mullany, Janet A Englund, Helen Chu, Steven C LeClerq, Joanne Katz

**Affiliations:** 1 Department of International Health, Johns Hopkins Bloomberg School of Public Health, 615 N. Wolfe Street, Room W5009, 21203-2105 Baltimore, MD USA; 2 Department of Global Health, Milken Institute School of Public Health, George Washington University, Washington, DC USA; 3 Nepal Nutrition Intervention Project, Sarlahi, Kathmandu, Nepal; 4 Johns Hopkins Bloomberg School of Public Health, Baltimore, MD USA; 5 Seattle Children’s Research Institute, University of Washington, Seattle, WA USA; 6 Department of Medicine, University of Washington, WA Seattle, USA

**Keywords:** Nausea and vomiting in pregnancy, adverse birth outcomes, pregnancy, birthweight, low birth weight, small for gestational age, preterm birth, Nepal

## Abstract

**Background:**

Nausea and vomiting are experienced by a majority of pregnant women worldwide. Previous studies have yielded conflicting results regarding their impact on birth outcomes and few studies have examined this relationship in settings with limited resources. We aimed to determine the effect of nausea, vomiting and poor appetite during pregnancy on birth outcomes in rural Nepal.

**Methods:**

Observational cohort study using data collected in two randomized, community-based trials to assess the effect of influenza immunization during pregnancy on reproductive and respiratory outcomes among pregnant women and their offspring. Pregnant women in Sarlahi District, Nepal were recruited from 2011 to 2013. Exposure was defined as nausea, vomiting or poor appetite at any point during pregnancy and by trimester; symptoms were recorded monthly throughout pregnancy. Adverse outcomes were low birth weight (LBW), preterm birth and small for gestational age (SGA). Adjusted relative risks (aRR) with 95% CIs are reported from Poisson regressions with robust variance.

**Results:**

Among 3,623 pregnant women, the cumulative incidence of nausea, vomiting or poor appetite was 49.5% (*n* = 1793) throughout pregnancy and 60.6% (*n* = 731) in the first trimester. Significantly higher aRRs of LBW and SGA were observed among women experiencing symptoms during pregnancy as compared to symptom free women (LBW: aRR 1.20; 95% CI 1.05 1.28; SGA: aRR 1.16; 95% CI 1.05 1.28). Symptoms in the first trimester were not significantly associated with any of the outcomes. In the second trimester, we observed significantly higher aRRs for LBW and SGA (LBW: aRR 1.17; 95% CI 1.01 1.36; SGA: aRR 1.16; 95% CI 1.05 1.29) and a significantly lower aRR for preterm birth (aRR 0.75; 95% CI 0.59 0.96). In the third trimester, we observed significantly higher aRRs for LBW and SGA (LBW: aRR 1.20; 95% CI 1.01 1.43; SGA: aRR 1.14; 95% CI 1.01 1.29).

**Conclusions:**

Symptoms of nausea, vomiting or poor appetite during pregnancy are associated with LBW, SGA and preterm birth in a setting with limited resources, especially beyond the first trimester.

**Trial registration:**

Prospectively registered at ClinicalTrials.gov on Dec 17, 2009 (NCT01034254).

## Background

Nausea and vomiting are experienced by 35–91% of pregnant women worldwide [1–10]. The consequences of nausea and vomiting for pregnant women correlate with the severity of symptoms and range from reduced quality of life and depressive symptoms to preeclampsia, malnutrition, weight loss and dehydration [4, 6, 11–14]. Severe symptoms such as nausea accompanied with vomiting have been suggested to have greater negative impact on maternal wellbeing, pregnancy outcomes as well as birth outcomes [4, 6, 15, 16]. There is a risk that women are being undertreated for nausea and vomiting during pregnancy due to the high prevalence and self-limiting nature of the condition as well as insufficient safety data for pharmacological treatment [13, 14, 17–19]. The etiology of nausea and vomiting of pregnancy is assumed to be multifactorial and sometimes considered an evolutionary response that protects the woman from ingesting harmful foods, which may further contribute to its undertreatment [14, 20]. One theory suggests that hormone levels, including human chorionic gonadotropin and estrogen, are responsible due to their concurrence with the peak of nausea and vomiting symptoms [6, 21, 22]. Additionally, factors associated with increased nausea and vomiting of pregnancy and hyperemesis gravidarum include lower education level, symptoms in a previous pregnancy, primigravity, obesity, younger age, family history of hyperemesis gravidarum, psychosocial morbidity and carrying a female fetus [4, 7, 12–14, 23].

Given the potential effects of nausea and vomiting on food intake and maternal well-being, intra-uterine growth restriction is a concern. Nausea and vomiting has been associated with lower-than-recommended weight gain in pregnancy, which in turn has been associated with small for gestational age infants (SGA) and prematurity [9, 12, 24, 25]. SGA is considered a measure of intrauterine growth restriction, as genetic differences in birth size are relatively small in healthy pregnancies [26]. The strength of association of nausea and vomiting in pregnancy with SGA, LBW and preterm birth are inconsistent in the literature [4, 7–9, 16, 25, 27–30]. Although inconclusive, it has been suggested that more severe symptoms of nausea and vomiting are more strongly associated with adverse birth outcomes [6, 15, 31].

Nausea and vomiting during pregnancy is especially concerning in low and middle income countries where resources might be limited in terms of food availability and access to health care, and where women may be nutritionally deficient prior to the start of pregnancy. Most prior studies have been based in high-income countries and few studies have examined this association in low-income settings [4, 7, 9, 16, 27, 29]. The objective of this study was to investigate the effects of nausea, vomiting and poor appetite during pregnancy on birth outcomes using data collected in the rural plains of southern Nepal.

## Methods

### Data collection

This is a secondary analysis of data collected in two sequential, randomized, community-based trials assessing the impact of maternal influenza immunization on reproductive outcomes, and incidence of influenza among pregnant women and their infants [32, 33]. During two 1-year periods in 2011–2013, married women of childbearing age were identified through a door-to-door census in the Sarlahi District of rural Nepal. The women were visited every 5 weeks and if they had not had a period since the past visit, they were offered a pregnancy test and consented for the trial. Two annual cohorts of pregnant women were eligible if they were married, 15–40 years of age, 17–34 weeks’ gestation at enrollment, and had not previously received any influenza vaccine that season. Women were excluded if they had already participated in an influenza study, did not intend to deliver their child in the study area, or were allergic to any vaccine component. For enrolled women, data collection at each visit included self-report of medical, surgical and reproductive history, tobacco use and several pregnancy-associated morbidities experienced in the past 30 days. Clinically collected health information at each study visit included weight, blood pressure, and pulse rate. Flowcharts describing the exact procedures in terms of inclusion criteria, exclusion criteria, and randomization for the original population are available in the original publications for these trials [32, 33].

### Study population

**Fig. 1:**
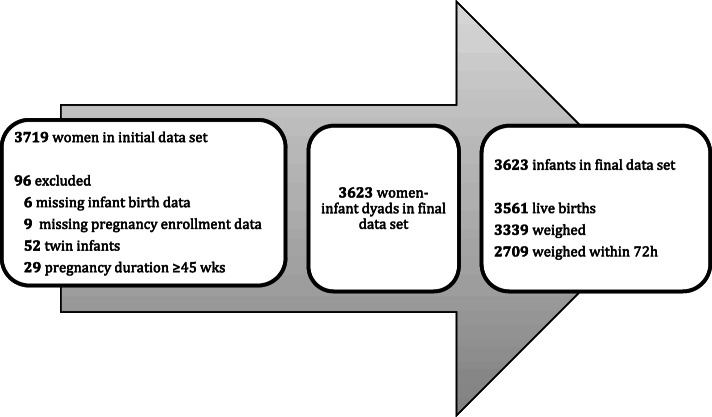
Flow chart showing the criteria used to reach the final analytical data set. Illustration of how we reached the number of women and infants included in the study based on the initial data set, the final number of women-infant dyads and number of infants with available birth outcome data

3623 women were included in this analysis. Women were excluded if they were not carrying singletons (*n* = 26) and if gestational age at birth calculated based on last menstrual period exceeded 45 weeks (*n* = 29) (Fig. 1). Gestational age at different time points (enrollment, monthly visits, and delivery date) was estimated using the difference in weeks between the date of last menstrual period and the time point of interest.

### Exposures

The symptoms of exposure included any self-reported nausea, vomiting and poor appetite during any of the 30 days prior to each visit. If a symptom was reported, the gestational age at which the symptom occurred was determined by calculating the difference in weeks between the midpoint of the 30-day symptom period, that is 15 days before the visit, and date of last menstrual period. We selected any reported nausea, vomiting or poor appetite throughout pregnancy and by trimester as the primary exposure. While nausea, vomiting and poor appetite are separate symptoms with potentially different levels of health consequences, all symptoms, whether in combination or alone, have the potential to affect nutritional status and gestational weight gain, which in turn may affect fetal growth and birth outcomes, especially SGA. The exposure was categorized and analyzed by trimester, which were defined as < 12 weeks (first trimester), 12–27 weeks (second trimester), and ≥ 27 weeks (third trimester). In any comparison between symptom groups related to trimester, we compared women with nausea, vomiting or poor appetite in a specific trimester with symptom-free women in the same trimester. If a woman had multiple visits during one trimester, the visits were grouped so that the exposure for that trimester was only registered once per woman. In this way, each woman only contributed to the exposure group once per trimester. For descriptive purposes, we also examined the proportion of women with a visit in each month of pregnancy as well as the cumulative incidence of nausea, vomiting or poor appetite in the women in that specific month of pregnancy.

Given that we wanted to look at preterm birth as an outcome, we restricted the exposure assessment to have occurred before 37 weeks. For descriptive purposes, we also examined the cumulative incidence overall and by trimester of different combinations of nausea, vomiting and poor appetite such as having all symptoms or just some of the symptoms throughout pregnancy or in a specific trimester. The combinations included nausea or poor appetite, vomiting or poor appetite, nausea or vomiting, poor appetite only, nausea only, vomiting only, nausea and vomiting, nausea and poor appetite, vomiting and poor appetite, as well as nausea and vomiting and poor appetite.

As an attempt to look at severity of symptoms, we examined the proportions of women who sought medical attention for their symptoms. Given that only 5.3% (*N* = 95) of women with nausea, vomiting or poor appetite sought medical attention, we determined that numbers were too low to include in the analysis.

### Outcomes

The primary outcomes included LBW (< 2500 g), preterm birth (< 37 weeks) and SGA (< 10th centile of the Intergrowth 21 reference standard) [26, 34]. Postnatal weight was considered birthweight if obtained within 72 h after birth. For infants born at > 42 weeks, the standard for gestational age of 42 weeks was used.

### Statistical analyses

Across all analyses, birth outcomes for women with any nausea, vomiting or poor appetite during pregnancy were compared to birth outcomes for women who had been symptom free throughout. Birth outcomes for women with nausea, vomiting and poor appetite in a given trimester were compared to birth outcomes for women who had been symptom free during that specific trimester.

T-tests and Pearson’s Chi-squared tests were performed to compare differences in maternal and infant characteristics across symptom groups as described above. A *p*-value below 0.05 was considered statistically significant across all statistical tests.

Poisson regression with robust variance and a 95% confidence interval (CI) was used to estimate the associations between the four exposures and the three outcomes. Covariates included age of mother in years (≤ 19, 20–24, 25–30, 30–35, ≥ 35), maternal education (education/no education), maternal parity (nulliparous/multiparous), smoking during pregnancy (yes/no) gestational age at birth (weeks), sex of infant (female/male) and number of study visits per woman. In the models with preterm birth as the outcome, gestational age at birth was excluded from the regression covariates.

## Results

**Fig. 2:**
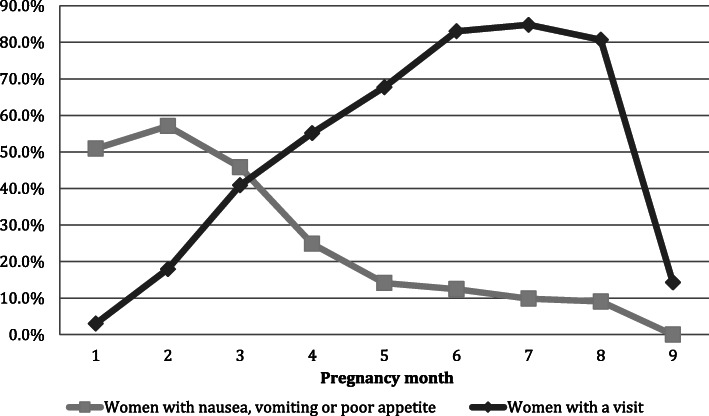
Visit data and symptoms of nausea, vomiting or poor appetite by pregnancy month. Proportion of women with at least one visit in a given pregnancy month and the cumulative incidence of symptoms of nausea, vomiting or poor appetite at any point during the corresponding pregnancy month

The cumulative incidence of any nausea, vomiting or poor appetite during pregnancy was 49.5% (*n* = 1793). When separated by trimester, the proportion of women who experienced NVP in each trimester was, (1) 60.6% (*n* = 731) of the 1206 women with a visit in the first trimester, (2) 34.2% (*n* = 1137) of the 3323 women with a visit in the second trimester, and (3) 15.2% (*n* = 517) of the 3401 women with a visit in the third trimester. The proportion of women with a visit varied by pregnancy month as did the cumulative incidence of nausea, vomiting or poor appetite (Fig. 2). The proportion of women with at least one visit in each month of pregnancy increased steadily throughout pregnancy except for the eight and ninth month when it started to decrease again. Conversely, the cumulative incidence of nausea, vomiting or poor appetite steadily decreased for every increase in month of pregnancy from beginning to end, with the exception of the first month of pregnancy, which had a frequency of symptoms that was slightly lower than the second month of pregnancy. The cumulative incidence of nausea, vomiting or poor appetite by pregnancy month ranged from 5.8% (ninth month of pregnancy before 37 weeks) to 57.1% (second month of pregnancy). The month where most women had a recorded visit was the seventh month of pregnancy at 84.8%, and the lowest proportion of women with a visit was recorded for the first month of pregnancy at 3%.

**Fig. 3:**
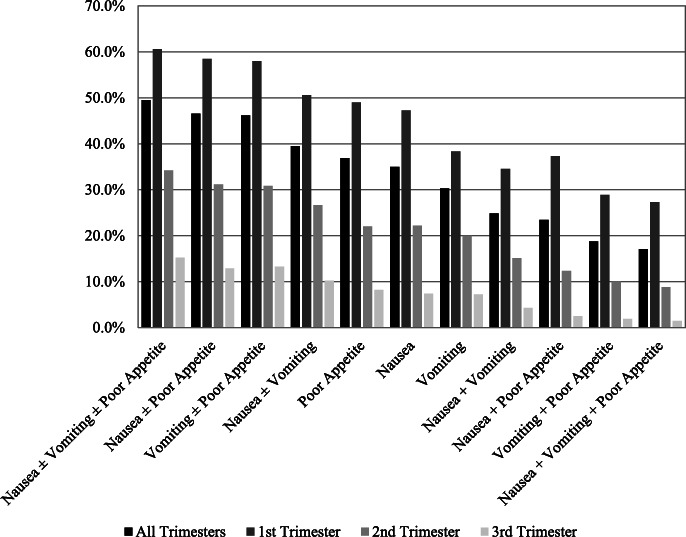
Symptom combinations throughout pregnancy and by trimester. Cumulative incidence of all combinations of nausea, vomiting and poor appetite throughout all trimesters of pregnancy and separated by trimester

When looking at the cumulative incidence of different combinations of nausea, vomiting and poor appetite such as the frequency of having all symptoms or just some of the symptoms throughout pregnancy and by trimester, we saw that the frequency of different symptom combinations decreased as the symptom combination became more restricted to include more symptoms (Fig. 3). A similar pattern in terms of decreasing cumulative incidence with increasing trimester was observed for all the symptom combinations.

The mean number of visits per woman, including the enrollment visit, was 5.3 (Table 1). Women with symptoms of nausea, vomiting or poor appetite overall and in each trimester had a significantly higher mean number of visits compared to symptom free women both overall and in each trimester. Women with symptoms on average had 1.2 more visits compared to women who were symptom-free throughout (5.9 visits vs. 4.7 visits; *p* < 0.001). The overall mean gestational age at enrollment was 17.6 weeks. Women with symptoms of nausea, vomiting or poor appetite were generally enrolled at an earlier mean gestational week of pregnancy as compared to symptom-free women (15.2 weeks vs. 20.1 weeks; *p* < 0.001). Similarly, statistically significant differences were observed when comparing the gestational age at enrollment in women with symptoms compared to without symptoms in each specific trimester. The majority of women were enrolled in the second trimester and 22.6% of women were enrolled in the first trimester. The mean age of the women in the study was 22.6 years, and this was similar across the symptom groups. Neither the mean age nor age groups differed significantly when comparing women with symptoms to symptom free women. About 40% of the women had no education and a similar proportion of women were illiterate. When comparing women with symptoms vs. without symptoms in the third trimester specifically, we observed a statistically significant lower proportion of literate women in the symptomatic group (53.8% vs. 62.4%; *p* < 0.001). Similarly, the mean number of education years differed significantly between women with and without symptoms in the third trimester specifically (4.4 vs. 5.1 years; *p* = 0.002). Nearly 2/3 of the women had given birth before and of these, 12.1% and 5.2% had experienced a previous spontaneous miscarriage and a previous stillbirth, respectively. Overall and in the third trimester, we observed a significantly higher proportion of women with a history of spontaneous miscarriage in symptomatic women (overall: 13.9% vs. 10.3%; *p* = 0.012; third trimester: 15.6% vs. 11.3%; *p* = 0.028). In addition, overall and in the third trimester, mean gravidity was significantly higher in symptomatic women vs. symptom-free women (overall: 2.3 pregnancies vs. 2.1 pregnancies; *p* < 0.001; third trimester: 2.4 pregnancies vs. 2.2 pregnancies; *p* < 0.001). Tobacco use at any point during pregnancy was reported in 4.1% of all women (*n* = 146), and this did not differ across symptom groups overall or by trimester. Receipt of influenza vaccine did not differ significantly across symptom groups except when looking at the second trimester, which showed that symptomatic women had more often received the influenza vaccine (52.6% vs. 48.7%; *p* = 0.034).

**Table 1:** Baseline demographic characteristics of the women separated by symptom group for nausea, vomiting or poor appetite before 37 weeks of pregnancy overall and by trimester

	Total (*n* = 3623)	SF (*n* = 1830)	NVP (*n* = 1793)	NVP1 (*n* = 731)	NVP2 (*n* = 1137)	NVP3 (*n* = 517)
	**Mean (SD)**					
Age	22.6 (4.7)	22.6 (4.7)	22.5 (4.7)	22.4 (4.4)	22.4 (4.9)	22.9 (4.9)
Gestational age	17.6 (7.0)	20.1 (6.8)	15.2 (6.3)	9.8 (2.3)	15.7 (5.3)	18.8 (7.1)
Gravidity	2.2 (1.5)	2.1 (1.4)	2.3 (1.5)	2.2 (1.4)	2.2 (1.5)	2.4 (1.7)
Years of education	5.0 (4.8)	5.1 (4.9)	4.8 (4.8)	5.3 (4.8)	4.8 (4.7)	4.4 (4.8)
Total Visits	5.3 (1.9)	4.7 (1.8)	5.9 (1.8)	7.1 (1.4)	5.8 (1.7)	5.3 (1.8)
	**N (%)**					
Age						
≤19	1021 (28.6)	507 (28.3)	514 (29.0)	213 (29.3)	336 (29.9)	135 (26.7)
20–24	1487 (41.7)	735 (41.0)	752 (42.4)	309 (42.6)	475 (42.3)	214 (42.3)
25–29	760 (21.3)	409 (22.8)	351 (19.8)	149 (20.5)	215 (19.1)	101 (20.0)
30–34	223 (6.3)	109 (6.1)	114 (6.4)	46 (6.3)	67 (6.0)	40 (7.9)
≥35	78 (2.2)	35 (2.0)	43 (2.4)	9 (1.2)	31 (2.8)	16 (3.2)
Gestational age						
<12	820 (22.6)	232 (12.7)	588 (32.8)	523 (71.6)	254 (22.3)	89 (17.2)
12–27	2298 (63.4)	1215 (66.4)	1083 (60.4)	208 (28.5)	844 (74.2)	333 (64.4)
≥27	505 (13.9)	383 (20.9)	122 (6.8)	-	39 (3.4)	95 (18.4)
Literate	2103 (60.6)	1076 (61.6)	1027 (59.7)	444 (63.3)	655 (59.9)	265 (53.8)
No education	1474 (42.5)	738 (42.3)	736 (42.8)	270 (38.5)	465 (42.6)	239 (48.6)
Smoking	146 (4.1)	76 (4.2)	70 (3.9)	15 (2.1)	45 (4.0)	29 (5.6)
Flu vaccine	1816 (50.1)	905 (49.5)	911 (50.8)	356 (48.7)	598 (52.6)	265 (51.3)
Nulliparous	1523 (42.1)	789 (43.1)	734 (40.9)	286 (39.1)	485 (42.7)	197 (38.1)
≥ 1 parity						
Previous miscarriage	254 (12.1)	107 (10.3)	147 (13.9)	65 (14.6)	85 (13.0)	50 (15.6)
Previous stillbirth	110 (5.2)	52 (5.0)	58 (5.5)	25 (5.6)	31 (4.8)	14 (4.4)

*SF Symptom free throughout pregnancy; NVP nausea, vomiting or poor appetite in any trimester; NVP1-3 nausea, vomiting or poor appetite in that specific trimester. Gestational age in weeks*

The mean gestational age at birth was 38.8 weeks and this was similar across all symptom groups (Table 2). Statistical differences in gestational age at birth were observed for overall symptoms as well as symptoms in the first and second trimester (overall: 39.0 weeks vs. 38.7 weeks; *p* = 0.013; first trimester: 38.8 weeks vs. 38.5 weeks; *p* = 0.046; second trimester: 39 weeks vs. 38.7 weeks; *p* = 0.003). The mean birthweight was slightly lower (39 g) for infants whose mothers any nausea, vomiting or poor appetite in pregnancy as compared to infants whose mothers had been symptom free throughout pregnancy (2768 g vs. 2807 g; *p* = 0.027). The difference in mean birthweight between infants of symptomatic women compared to symptom-free women increased by trimester of exposure. There was a 20 g difference for NVP in the first trimester (*p* = 0.495), 38 g for second trimester exposure (*p* = 0.047), and 86 g for third trimester exposure (*p* = 0.002). About 75% (*n* = 2709) of infants had available birth outcome data in terms of weight, length and head circumference. Approximately 24% (*n* = 661) of the infants were LBW while 37% (*n* = 1011) were SGA. The incidence of preterm birth was 13.5% and 1.7% of infants were stillborn. A larger proportion of infants had LBW if the mother had experienced nausea, vomiting or poor appetite overall and during each trimester of pregnancy; however, the difference was only statistically significant during the third trimester of pregnancy (NVP3: 29.6% vs. SF3: 23.3%; *p* = 0.007). The proportion of SGA infants was significantly higher among symptomatic than symptom-free women overall and for the second and third trimesters (overall: 40.1% vs. 34.6%; *p* = 0.003; second trimester: 41.7% vs. 34.0%; *p* < 0.001; third trimester: 44.0% vs. 36.7%; *p* = 0.006). For preterm birth, symptoms at any point during pregnancy before 37 weeks yielded 12.6% preterm infants, whereas in the symptom-free group, the number was 14.5%, however, the difference was not statistically significant (*p* = 0.089). For women with symptoms in the first and second trimesters, however, significantly higher proportions of preterm births were seen comparing to symptom-free women in the same trimesters (first trimester: 13.1% vs. 18.3%; *p* = 0.014; second trimester: 12.1% vs. 14.8%; *p* = 0.034). Symptomatic women had a lower proportion of stillbirths compared to symptom-free women (1.3% vs. 2.1%; *p* = 0.049). The proportion of female infants was slightly higher if the mother had experienced symptoms of nausea, vomiting or poor appetite overall and in the first and third trimester; however, none of the differences were statistically significant (overall: 48.1% vs. 46.8%; *p* = 0.432).

**Table 2:** Infant birth characteristics separated by symptom group for nausea, vomiting or poor appetite during pregnancy

	Total (*n* = 3623)	SF (*n* = 1830)	NVP (*n* = 1793)	NVP1 (*n* = 731)	NVP2 (*n* = 1137)	NVP3 (*n* = 517)
	**Mean (SD)**					
Gestational age	38.8 (2.7)	38.7 (2.6)	39.0 (2.7)	38.8 (2.5)	39.0 (2.9)	39.2 (2.3)
Weight	2788 (451)	2807 (452)	2768 (450)	2782 (436)	2769 (447)	2716 (480)
Length	47.8 (2.2)	48.0 (2.1)	47.7 (2.2)	47.6 (2.1)	47.7 (2.3)	47.6 (2.2)
Head circumference	32.9 (1.6)	33.0 (1.5)	32.8 (1.8)	32.7 (1.7)	32.8 (1.8)	32.7 (1.7)
Female	1717 (47.4)	855 (46.8)	862 (48.1)	355 (48.6)	538 (47.4)	252 (48.7)
LBW	661 (24.4)	316 (23.2)	345 (25.6)	139 (25.1)	209 (24.9)	116 (29.5)
SGA	1011 (37.3)	470 (34.6)	541 (40.1)	214 (38.6)	350 (41.7)	173 (44.0)
Preterm	490 (13.5)	265 (14.5)	225 (12.6)	96 (13.1)	138 (12.1)	57 (11.0)
Stillbirth	62 (1.7)	39 (2.1)	23 (1.3)	6 (0.8)	20 (1.8)	5 (1.0)

*SF Symptom free throughout pregnancy; NVP nausea, vomiting or poor appetite in any trimester; NVP1-3 nausea, vomiting or poor appetite in that specific trimester; LBW low birth weight (< 2500 g); SGA small for gestational age; Preterm born < 37 weeks’ gestation. Gestational age in weeks*

After adjusting for maternal age, smoking during pregnancy, parity, education, sex of the infant, gestational age at birth, vaccine status and number of visits, the risk of LBW for symptomatic women was 20% higher compared to women who had been symptom-free throughout pregnancy (aRR 1.20; 95% CI 1.05 1.38) (Table 3). When comparing symptomatic to asymptomatic women in each trimester, the increased risk of LBW only remained statistically significant in women experiencing symptoms the second (aRR 1.17; 95% CI 1.01 1.36) and third trimester (aRR 1.20; 95% CI 1.01 1.43). The adjusted relative risk for LBW in women with symptoms in the first trimester was 1.10 (95% CI 0.87 1.39).

**Table 3:** Incidences, unadjusted and adjusted relative risks for birth outcomes in relation to symptom group and trimester for nausea, vomiting or poor appetite during pregnancy

ALL TRIMESTERS				
		**N (%)**	**Unadjusted RRs (95% CI)**	**Adjusted RRs (95% CI)**
LBW	SF (Ref)	316 (23.2)	1.00	1.00**
	NVP	345 (25.6)	1.10 (0.96–1.26)	1.20 (1.05–1.38)*
SGA	SF (Ref)	470 (34.6)	1.00	1.00**
	NVP	541 (40.1)	1.16 (1.05–1.28)*	1.16 (1.05–1.28)*
Preterm birth	SF (Ref)	265 (14.5)	1.00	1.00***
	NVP	225 (12.6)	0.87 (0.73–1.02)	0.87 (0.70–1.09)
**FIRST TRIMESTER**				
		**N (%)**	**Unadjusted RRs (95% CI)**	**Adjusted RRs (95% CI)**
LBW	SF1 (Ref)	90 (24.2)	1.00	1.00**
	NVP1	139 (25.1)	1.04 (0.82–1.3)	1.10 (0.87–1.39)
SGA	SF1 (Ref)	131 (35.2)	1.00	1.00**
	NVP1	214 (38.6)	1.09 (0.92–1.30)	1.10 (0.93–1.31)
Preterm birth	SF1 (Ref)	87 (18.3)	1.00	1.00***
	NVP1	96 (13.1)	0.72 (0.55–0.94)*	1.10 (0.84–1.44)
**SECOND TRIMESTER**				
		**N (%)**	**Unadjusted RRs (95% CI)**	**Adjusted RRs (95% CI)**
LBW	SF2 (Ref)	385 (25.5)	1.00	1.00^a^
	NVP2	209 (24.9)	1.06 (0.91–1.22)	1.17 (1.01–1.36)
SGA	SF2 (Ref)	556 (34.0)	1.00	1.00^a^
	NVP2	350 (41.7)	1.23 (1.10–1.36)*	1.16 (1.05–1.29)*
Preterm birth	SF2 (Ref)	324 (14.8)	1.00	1.00^b^
	NVP2	138 (12.1)	0.82 (0.68–0.99)*	0.75 (0.59–0.96)*
**THIRD TRIMESTER**				
		**N (%)**	**Unadjusted RRs (95% CI)**	**Adjusted RRs (95% CI)**
LBW	SF3 (Ref)	525 (23.3)	1.00	1.00^a^
	NVP3	116 (29.5)	1.26 (1.07–1.51)*	1.20 (1.01–1.43)*
SGA	SF3 (Ref)	828 (36.7)	1.00	1.00^a^
	NVP3	173 (44.0)	1.20 (1.06–1.36)*	1.14 (1.01–1.29)*
Preterm birth	SF3 (Ref)	340 (11.8)	1.00	1.00^b^
	NVP3	57 (11.0)	0.94 (0.72–1.22)	1.14 (0.88–1.47)

RR relative risk; LBW low birth weight (< 2500 g); SGA small for gestational age; Preterm birth = born < 37 weeks’ gestation; SF symptom free throughout pregnancy; SF1-3 symptom free in the trimester corresponding to the number; NVP nausea, vomiting or poor appetite in any trimester; NVP1-3 nausea, vomiting or poor appetite in each trimester separately. *Statistically significant at alpha 0.05.^*a*^*Adjusted for mother’s age, smoking during pregnancy (any time: yes/no), parity (0/≥1), education (yes/no), sex of infant (male/female), gestational age at birth, vaccine status and number of visits.*^b^*Adjusted for mother’s age, smoking during pregnancy (any time: yes/no), parity (0/≥1), education (yes/no), sex of infant (male/female), vaccine status, number of visits*

After adjustment for covariates, the risk of SGA for symptomatic women was 16% higher compared to women who had been symptom-free throughout pregnancy (aRR 1.16; 95% CI 1.05 1.28). When comparing symptomatic to asymptomatic women in each trimester, the increased risk of SGA only remained statistically significant in women experiencing symptoms the second (aRR 1.16; 95% CI 1.05 1.29) and third trimester (aRR 1.14; 95% CI 1.01 1.29). The adjusted relative risk for SGA comparing women with and without symptoms in the first trimester was 1.10 (95% CI 0.93 1.31).

For preterm birth, gestational age at birth was excluded from the covariates in the regression. In the overall and second trimester analysis, experiencing symptoms of nausea, vomiting and poor appetite were associated with a tendency to reduced risk of preterm birth, however, the association was only statistically significant when comparing symptomatic to asymptomatic women in the second trimester (aRR 0.75; 95% CI 0.59 0.96). The adjusted relative risk for preterm comparing women with and without symptoms throughout pregnancy was 0.87 (95% CI 0.70 1.09). In the first and third trimester, symptoms of nausea, vomiting and poor appetite were associated with a slightly increased risk for preterm birth compared to symptom free women, however, the results were not statistically significant (first trimester: aRR 1.10; 95% CI 0.84 1.44; third trimester: aRR 1.14; 95% CI 0.88 1.47).

## Discussion

This observational cohort study showed that symptoms of nausea, vomiting or poor appetite during pregnancy were associated with adverse birth outcomes. Overall, symptoms experienced any time during pregnancy was significantly associated with a 20% increased risk of LBW and a 16% increased risk of SGA. Symptoms during the second trimester were significantly associated with all the outcomes and showed a 17% increased risk of LBW, a 16% increased risk of SGA and a 25% reduced risk of preterm birth. In the third trimester, symptoms were significantly associated with a 20% increased risk for LBW and a 14% increased risk for SGA. Symptoms during the first trimester were not significantly associated with any of the outcomes.

Previous literature lacks consensus about whether nausea or vomiting in pregnancy increases the risk of LBW. Studies in low-income countries have generally been in agreement with our results [8]. Some studies from middle-to-high-income settings have not reported positive associations [4, 7, 9, 27]. This suggests that it might be the combination of symptoms and setting (e.g. maternal malnutrition) that leads to adverse birth outcomes, rather than the symptoms themselves. The conflicting results may also be due to differences in classification of exposure.

The finding that symptoms in the first trimester are not associated with adverse birth outcomes suggests that symptoms during later trimesters in pregnancy may be more severe in terms of its adverse effects on the fetus. This has not been shown in the previous literature. A previous study showed an association between nausea and vomiting in late pregnancy and lower birth weights in the infant and lower weight gain in the mother, and that the effect on birth weight was even more significant when the weight gain was poor in the mother [35]. Additionally, women with hyperemesis gravidarum more often have persisting symptoms throughout pregnancy, and hyperemesis gravidarum has in turn been associated with adverse birth outcomes [16, 21]. This agrees with our study findings that nausea, vomiting or poor appetite experienced in mid to late pregnancy have higher relative risks of adverse birth outcomes than earlier in pregnancy. It also raises the question of whether our findings could reflect an additive effect of symptoms across all trimesters since women with symptoms in the second and third trimester might be more likely to have experienced symptoms in the earlier trimesters as well. One study showed that severe early pregnancy vomiting was associated with vomiting in the third trimester, and that this had a greater impact on maternal nutritional intake and infant birthweight [31]. Another study showed an increased risk of SGA in women who had hyperemesis gravidarum during pregnancy, but studies on the effects of milder symptoms have shown either a protective effect of nausea and vomiting of pregnancy or no difference [4, 29, 30]. Of note, these studies all took place in high-income countries. On the other hand, studies in high-income settings have showed a significantly increased risk of LBW and SGA in women with hyperemesis gravidarum [16, 29].

There was a lower adjusted relative risk of preterm birth among women with nausea, vomiting or poor appetite in the second trimester compared to symptom free women when one of the covariates adjusted for was number of visits per woman. We included number of visits as a covariate given that some women may have had fewer visits because they had a preterm birth. With fewer visits, the chance of capturing the experience of symptoms during pregnancy would also have decreased. Equivocal results around preterm birth have been observed in previous studies. However, no studies to date have examined the effect of nausea, vomiting or poor appetite during pregnancy on preterm birth in a low-income, rural setting as in this study [4, 23, 25, 28, 29].

The present study showed a cumulative incidence of nausea, vomiting or poor appetite of 60.6% during the first trimester, which is on the lower end of what has been previously reported [1, 5]. Previous literature has shown that nausea and vomiting during pregnancy is less likely to be reported among Asian and African populations as compared to Caucasian populations and the cumulative incidence in the current study agrees with previously reported numbers from Bangladesh and Tanzania [2, 8, 13]. On the other hand, hyperemesis gravidarum has been shown to be more commonly diagnosed among women of Asian ethnicities as compared with Caucasians, which may contribute to why the present study showed positive associations between nausea, vomiting or poor appetite and adverse birth outcomes in the light of other studies having failed to do so [21, 22].

Strengths of the study include detailed population-based data on obstetric history, pregnancy morbidity and infant birth characteristics in a large number of mother-infant pairs. The prospective nature of the data ensured that temporality was not an issue and minimized the risk of recall bias. Since the women were interviewed monthly, we ensured that the recall time was relatively short for both the date of last menstrual period and reported symptoms.

Limitations include that while nausea and vomiting is most common during the first trimester, relatively few women were enrolled in the first trimester (22.6%) due to the enrollment protocol. Therefore, for most women we only had information from the second and third trimesters. Nausea and vomiting of pregnancy peaks during the first trimester and is uncommon after 22 weeks gestation [7, 9, 10]. However, given that we were able to analyze the data by trimester, we do not perceive this as a major problem unless there were some confounders that might have been associated with both first trimester enrollment and the exposure. Also, because few women enrolled in the first trimester we were unable to calculate weight gain during pregnancy or pre-pregnancy body mass index (BMI). Weight gain and BMI can affect both the development of nausea, vomiting and poor appetite as well as birth outcomes. Obesity has been shown to increase the risk of nausea and vomiting during pregnancy and has also been associated with the risk of adverse birth outcomes [36–38]. Additionally, inadequate weight gain during pregnancy has been linked to adverse birth outcomes including LBW, SGA and preterm birth [12, 24]. Nausea and vomiting, in particular severe vomiting such as in hyperemesis gravidarum, has been linked to inadequate weight gain and potentially even weight loss [12, 13, 16, 17, 25]. If we had information on weight at the start of pregnancy, we might also have been able to detect weight loss and, with that, potentially detect hyperemesis gravidarum in the enrolled women. Future studies should attempt to design data collection so that it allows for pre-pregnancy or early first trimester BMI and weight to be collected. We also did not collect data on severity of symptoms during pregnancy. Other studies have shown a difference in the effect of mild symptoms of nausea vs. more severe symptoms of repeated vomiting in the form of hyperemesis gravidarum [4, 29, 31]. For example, severe nausea and vomiting (defined as not being able to retain meals) has been associated with reduced food intake to a higher degree than milder symptoms [39]. In addition, another study showed that vomiting associated with lower birthweight as opposed to nausea alone if symptoms were experienced in the first trimester [15]. While we had number of days in the past 30 days where each symptom was present, the time at risk for exposure by trimester or total pregnancy was variable, depending on when they enrolled and length of pregnancy. In addition, the number of days of symptoms was not differentiated by when in the past 30 days these had occurred and whether the symptoms overlapped in time or were experienced at distinct time periods. Therefore we were unable to isolate symptoms and examine duration of exposure more precisely. We attempted to examine the severity of symptoms by using seeking medical attention for symptoms as a proxy for severity, but given that only 5.3% (*N* = 95) of exposed women sought medical attention for the symptoms, we determined that numbers were too low to include in the analysis. Another limitation was the inability to distinguish between nausea, vomiting or poor appetite due to pregnancy vs. other causes. Nausea, vomiting and poor appetite during pregnancy may have variable etiologies that may need to be considered and grouping the symptoms together may have further complicated this issue [7, 10]. It may have been useful to collect symptom patterns in terms of onset (few women start having symptoms after 9 weeks) and temporal patterns (nausea and vomiting of pregnancy may be more persistent and continuous across weeks and persistent throughout the day) or, as other studies have done, separate women who reported nausea and vomiting in association with fever or diarrhea [14, 18]. Sample size might also have been an issue when estimating the effect size of nausea, vomiting or poor appetite during pregnancy by trimester as fewer women reported symptoms in the second and third trimester.

Despite these limitations, the results suggest nausea, vomiting or poor appetite during pregnancy in this limited resource setting have a significant impact on birth outcomes, particularly in the second and third trimesters. These symptoms are often considered to be normal in pregnancy given that they are so common and generally self-limiting, but the effects of such symptoms in settings where resources and access to health care are limited need to be examined [28, 39–41]. Several studies have shown that women with nausea and vomiting of pregnancy tend to change their diet during pregnancy and steer away from certain foods [14, 17]. Limited resources may affect the woman’s ability to adjust her diet accordingly. These results challenge the notion that nausea and vomiting are harmless symptoms of pregnancy, which can be used to raise awareness among pregnant women and health care workers in these settings. Of note, limited resource settings are not confined to rural areas of developing countries, which is where this study took place. Attention should be given as well to these issues in urban parts of developing countries and potentially in certain areas of higher income countries, which may be highly affected by poverty and health disparities as well.

While the evidence for efficacy is currently limited, there are several accepted treatments for nausea and vomiting that are considered safe in pregnancy. These include pre-conception vitamin supplementation, dietary changes, pharmacologic treatment with antiemetics or vitamin B6, and intravenous fluid replacement [42]. In addition, studies have shown that treatment of early symptoms may prevent later complications [14, 17, 42]. In terms of pharmacologic treatment, several medications are considered safe and effective. These include vitamin B6 supplementation with or without doxylamine, which is considered first line pharmacologic treatment in the United States, and dopamine antagonists such as metoclopramide [43, 44]. While some of these interventions may not be suitable for low resource settings, nutritional support, pre-conception vitamin supplementation, and oral rehydration therapy could be considered relatively inexpensive interventions in terms of reducing the impact of nausea and vomiting in these settings. Micronutrient deficiencies and limited access to adequate nutrition is a significant concern in developing countries. Given this, targeted interventions in low resource settings may have an even greater benefit on reducing the impact of nausea, vomiting and poor appetite in pregnancy, including reducing the impact of milder symptoms that would not have been medically treated in a high income setting.

## Conclusions

Pregnant women experiencing nausea, vomiting or poor appetite during pregnancy in a low resource setting have an increased relative risk of LBW and SGA and a decreased relative risk for preterm birth. The estimates differ by trimester in which the symptoms were experienced. Specifically, symptoms in the second and third trimester have the most impact on the studied birth outcomes. Further studies are needed in similar settings and where symptom severity as well as pre-pregnancy BMI is available. Given that inadequate nutrition and limited access to vitamin supplements is common in low resource settings, interventions targeting these issues should be explored as a way to reduce the impact of nausea, vomiting and poor appetite in high-risk populations.

## Data Availability

The datasets generated and/or analyzed during the current study are available from Dr. Joanne Katz upon reasonable request.

## Abbreviations

SGA: Small for gestational age
LBW: Low birth weight
BMI: Body mass index
